# RETRACTED: Abdullah, M.M.S.; Al-Lohedan, H.A. Novel Bio-Based Amphiphilic Ionic Liquids for the Efficient Demulsification of Heavy Crude Oil Emulsions. *Molecules* 2021, *26*, 6119

**DOI:** 10.3390/molecules31203603

**Published:** 2026-10-10

**Authors:** Mahmood M. S. Abdullah, Hamad A. Al-Lohedan

**Affiliations:** Department of Chemistry, College of Science, King Saud University, P.O. Box 2455, Riyadh 11451, Saudi Arabia; hlohedan@ksu.edu.sa

The journal retracts the article “Novel Bio-based Amphiphilic Ionic Liquids for the Efficient Demulsification of Heavy Crude Oil Emulsions” [1] cited above.

Following publication, concerns were brought to the attention of the Editorial Office regarding the integrity of the figures presented in the article [1].

In accordance with the journal’s standard procedures, the Editorial Office and Editorial Board conducted an investigation, which confirmed indications of inappropriate editing within Figure 5 and duplication between Figure 8 in this article [1] and Figure 8 in previously published material by a similar authorship group [2], presenting different experimental conditions. Although the authors cooperated during the investigation, the Editorial Board concluded that the concerns identified could not be satisfactorily resolved based on the information and materials provided. As a consequence, the Editorial Board lost confidence in the reliability of the findings presented in this article and has decided to retract this publication in accordance with MDPI’s retraction policy.

This retraction was approved by the Editor-in-Chief of the journal *Molecules*.

The authors have been informed of this retraction and have indicated their disagreement with this decision.
